# Supplementary material for: Testing the Accuracy of Wearable Technology to Assess Sleep Behaviour in Domestic Dogs: A Prospective Tool for Animal Welfare Assessment in Kennels
Source: Animals (Basel). 2023 Apr 26;13(9):1467. doi: 10.3390/ani13091467 (PMC10177158; doi:10.3390/ani13091467)
Supplement: Supplementary file 1 [file animals-13-01467-s001.zip › animals-2310502-supplementary.pdf]

Supplementary Material 1

Table 1. Definition of behavioural categories used to assess diurnal and nocturnal behaviours of dogs.

Behavioural states		Definition
**Consumption**	Feed	Dog is actively consuming food.
	Drink	Dog is actively consuming water.
**Interaction**	Explore	Dog interacts with the environment, approaching stimuli in an investigative manner (smell, scratch, lick, touch, or dig).
**Locomotion**	Locomotion	Dog is moving from one location to another with alternate limbs touching the ground at different times throughout at any velocity.
**Rest**	Resting	Dog is laying down, stationary, with abdomen touching the ground with either dorsal, lateral, or caudal side. Limbs can be stretched in the front, laterally or curled near the body. Eyes open and movement of ears can be observed.
	Sleep	Dog has similar positions as resting, but eyes remain closed for at least 02 minutes. Paddling of limbs, rapid eye movement and occasional vocalizations can be expressed.
**Stationary**	Alert	Dog is in an upright position with all limbs extended. Ears are in upright position. Ears and head are pointing into the direction of stimuli.
	Sitting inactive	Dog is in an upright position, with hind legs flexed while front legs are extended straight.
	Stand inactive	Dog is in upright position with all limbs extended supporting the body while immobile.
**Social**	Social Affiliative	Dog is interacting (rub, smell other, present or groom other) with a conspecific in an affiliative manner.
	Social Aggressive	Dog is interacting (bite, chase, strike with paw, show teeth, hair bristle) with a conspecific in an aggressive manner.
	Play	Dog engages with a conspecific, less than two body lengths away, and different behaviours are observed (jump over other, chase, pulling tail, mouth, roll).
**Maintenance**	Groom	Dog grooms itself using its tongue, teeth, or mouth.
	Excrete	Dog releases faeces or urine from the body.
**Repetitive**	Tail-chasing	A quick and repetitive circular motion, where the dog attempts to bite its tail.
	Pacing	A locomotor movement with the animal traversing the same pathway at least twice in quick succession.
**Panting**		Dog's mouth is open, tongue out. Dog quickly inhales and exhales through mouth breathing. Chest breathing movements are visible.
**Vocalization**	Rhythmic Barking	Dog opens and closes mouth while emitting repetitively short and constant vocalizations that can vary in frequency from very high to deep and low.
